# Atlanta Society of Medicine

**Published:** 1896-02

**Authors:** W. L. Champion

**Affiliations:** Secretary; Atlanta


### *SOCIETY REPORTS.*

---

### ATLANTA SOCIETY OF MEDICINE.

ATLANTA, January 7, 1896.

Society was called to order by the President, Dr. Hurt. Members present were: Drs. Hurt, Champion, Duncan, Grandy, Hubbard, Dunbar Roy, Westmoreland, Stockard, Felder, Love, and Thomas.

The regular order for the evening was a discussion upon Typhoid Fever. The President requested Dr. Grandy to discuss the *diagnosis* of the disease.

DR. GRANDY said: "I did not expect, Mr. President, to be called up on such short notice. However, in many cases there will be little difficulty in making a diagnosis of typhoid, particularly if they present anything like the classic train of symptoms so beautifully described in the books. Unfortunately such typical cases are not common, and from the absence of certain of these symptoms and possibly the presence of others not usually associated with typhoid, serious doubt will often arise in the mind of the physician as to the actual condition present. This is especially true, of course, during the first week of the disease. In this section the diseases which most commonly have to be differentiated from typhoid are pneumonia and the so-called typho-malarial fever The form of pneumonia in question is one in which there is such depression of the vital powers that the patient is said to be in the typhoid condition. Here the history of the case, with the physical examination of the chest, the cough, and expectoration, ought to clear up the diagnosis. In my own experience my greatest difficulty has been to draw the line between cases of true typhoid and those of the so-called typho-malarial fever. A positive diagnosis cannot always be made within the first few days between these two conditions, and it cannot be foretold with certainty which of the two will yet develop. After the appearance of the rash, the abdominal soreness and tympanites, and the onset of nervous symptoms and the failure of quinine to control the temperature, the case is usually made out. I am aware that some physicians question the existence of typho-malaria; they claim that these cases are either typhoid or malaria, and there seems to be a disposition to class them as typhoid. The objection, I think, applies more to the name than to the condition. There certainly does exist in this part of the country a disease which is something more than intermittent or remittent malaria and something less than typhoid. Unfortunately for science there have been few autopsies to tell us whether these cases have the lesions of typhoid. I think the systematic use of Ehrlich's test for typhoid and the careful examination of the blood for the malarial organisms would throw much light on this subject, if they were more frequently applied."

DR. LOVE: "We are all familiar with the symptoms of typhoid fever. When consulted by patients in the first stages they complain of a dull, languid feeling in the morning, and more or less languid in the day, and as the disease progresses the temperature will rise, and usually get high in the afternoon, and tympanitic symptoms will appear, and soreness across the abdomen and in the iliac region. My experience has been that we meet with constipation more than with diarrhea. In some cases of typhoid fever there will be nervous symptoms and delirium. These usually have diarrhea and passage of blood from the bowels. When called to a case I prescribe calomel in small doses, and often repeat, and then continue the treatment with minute doses of phenacetin, bisulphate of quinine, and salol, and after the patient has taken to bed, and the temperature rises, I leave off the quinine, especially if the nervous form develops. I want to say here that I have treated in my whole practice in Atlanta but few cases of typhoid fever that did not have complications of malarial fever. I follow the above treatment with turpentine emulsion, and when the fever gets high, I use tincture of digitalis. In tympanitic condition, I use the turpentine enema, and I give milk and liquid beef as supportive treatment. To act on the bowels, I employ castor oil in preference to calomel. It acts better and softens the feces. Where there is any tendency to diarrhea I use opium, salol, and subnitrate of bismuth. I use the opium guardedly in typhoid fever, as too free use leads to brain complications. In pneumonic complications I treat as simple pneumonia. For reducing the temperature, there is nothing better than the pack, using wet sheets. All through the case keep up the supportive treatment, and treat all complications as you would the regular disease."

The President appointed DR. STOCKARD to give the *treatment*, and he spoke as follows : " In the treatment, I think that probably the world over, the most usual beginning is calomel, sometimes combined with ipecac ; and in this country, that is, in the South, it is the custom to give quinine in large doses before the diagnosis is entirely made out. After trying quinine and finding no effect on the fever, the usual course, probably, is to give turpentine emulsion. I think the best plan is to keep down the temperature with water, either with tub or sheet bath, or in some cases where they have an aversion to baths, is to reduce the temperature by a cold enema. I do not favor the administration of the coal-tar derivatives in typhoid fever, as we should harbor the strength of the patient as much as possible, and therefore they should not be used to lower the temperature, as it can be accomplished just as well with water, and with no bad effect on the patient, but will have a good tonic effect. The water stimulates the organs to their natural functions, and sustains the patient better than any other way. I think the calomel in the beginning, is important, as it puts the secretions in good condition, and I repeat it whenever necessary. I have never felt any fear in giving a dose, even in the second or third week, but would not give a large dose, simply one-fourth or one-sixth of a grain every few hours will have the effect without any depression and without any amount of peristalsis. Dr. Woodbridge has a treatment consisting of a combination of guaiacol, thymol, and eucalyptol, by which he claims to very much shorten the course of typhoid fever. I have used a modification of this treatment. Instead of giving small doses at short intervals, I have given large doses at intervals of three or four hours. In view of this discussion tonight I have jotted down some notes of cases that I have treated this way since last spring.

"A young man, aged nineteen, was taken three or four days before I saw him, and he had a temperature of from 101° to 102°. He had taken quinine and was feeling bad, had red tongue and tympanitic condition of the abdomen. I put him on the Woodbridge treatment, and a few days after his condition reached normal.

"The next case was a child five years of age and which I am sure was typhoid fever. When I first saw the case it had a temperature of 104°, and had been sick for some days. That evening the temperature reached 105°, and the tongue was heavily coated and the abdomen distended. I began with the calomel and used the sponge and sheet bath also. The fever gradually declined and also the tympanitic condition of the abdomen, and reached normal condition on the thirteenth day of the disease.

"Man, thirty years of age, who had been feeling bad for a week or more, and when I saw him had a temperature of 103°, with considerable uneasiness in the bowels and a rise of fever. I began the treatment on May 20th, and the next morning his temperature was $100\frac{2}{5}$°; on the 23d was $99\frac{1}{2}$°, and on the 26th was 99°.

"On October 16th was called to see a man who had a perfect case of typhoid fever with a temperature of 101°. I put him at once on this treatment, and his temperature was normal on the 21st.

"Case of an actress who had been sick three or four weeks when I was called. She had fever every night and had been taking phenacetin and continuing to work. In the morning her temperature was 104° and in the evening 105°, and had coated tongue, rash, and distended abdomen. On this treatment the abdominal symptoms subsided and the fever declined steadily.

"Where the poison has spent its force this treatment will hardly have any effect with the shortening of the course of the disease. In all these cases where temperature reached 102° it was reduced by cold sheet, sponge, or tub bath. I attribute more to the calomel than to the guaiacol. I think that to begin with calomel and go through with this treatment, using the baths to lower the temperature, typhoid will be favorably affected by it."

DR. FELDER: "I have just passed through an attack of typhoid fever, and would say something about it. I want to warn against the use of phenacetin in the third or fourth week when the temperature gets high, and it does not seem to be affected by the baths. My temperature would run up to 105° or 106°, and I would take one grain of phenacetin. This would cause profuse perspiration and a cold, clammy condition. I would never give it in such cases. In regard to the mode of giving baths, I would state that I had a patient who was very nervous and the preparations for giving the bath would worry her so as to run her temperature up two or three degrees, and it was necessary to give the bath every two hours, as otherwise her temperature would remain at from 103° to 106°. In her case I would put a pint of alcohol in the volume of water with cracked ice and would wet towels and wrap her body with these wet towels. I would let the towels remain for half an hour, until they got hot and the temperature would go down. I kept this up for two weeks, and at the same time paid most attention to the nourishment. She could not take sweet milk and I gave her buttermilk every two hours, also peptones and brandy. These baths were my treatment, except in the latter part of the disease her pulse became weak and quick, and I gave digitalis to keep up the strength. I also gave turpentine emulsion. She retained her strength remarkably well in such a marked case of typhoid fever."

DR. DUNCAN: "I do not give phenacetin in the last stages of typhoid fever. When you do not know what is the matter, and not positive about the fever, it might be given in the first week. The bowel symptoms in typhoid fever vary; in one case you will have constipation and in another diarrhea. For constipation in the first week or eight days I use one dram of boric acid and ten grains of sal soda in a gallon of water, and for diarrhea, ten grains of salol and ten grains of bismuth. For tympanitic condition give three grains of salol and two grains of sulphide of sodium every few hours. These will control most cases. I am in favor of giving calomel in the early stages. To support the heart I give veratrum, say, two drops of veratrum, three drops of gelsemium, and one drop of oil of cinnamon every three hours. In many cases the heart wears itself out. This regulates the temperature and improves the heart's action, the patient rests better, and the secretions improve. I always give small doses of deodorized tincture of opium. I am in favor of giving the patient as much buttermilk as he wants. It is better than sweetmilk, as the sweetmilk often forms a coagulum in the stomach. I do not give the turpentine emulsion. I use the turpentine stupe on the abdomen, but I use salol and sulphide of soda to control the tympanites. Liquid peptonoids are good. I leave off the solid food and use fluid diet, and get the patient not to eat solid food for three or four days after the fever has disappeared entirely. The delirium is benefited by fluid extract of gelsemium or bromide of sodium. Until I came to Atlanta hemorrhages from the intestines never gave me any trouble. I had a number of patients who had hemorrhages from the bowels, and they recovered, but when I came to Atlanta they were different. I have seen a few cases that recovered here in Atlanta that had profuse hemorrhages. I remember a young man who came from Florida and had typho-malarial fever. I assisted Dr. Stephons in that case. The young man had three profuse hemorrhages, and recovered, but as a rule they die here in Atlanta. I have tried various things. I have tried injections of acetate of lead, large doses of tincture of iron, bismuth, also large doses of charcoal. My success with hemorrhages in Atlanta has not been good."

DR. WESTMORELAND: "Typhoid fever always impressed me that it was a specific trouble, and as soon as I have recognized the case as typhoid I have recognized that there is more or less ulceration of the intestine, and a disease, as far as we know, that runs its course. There is no plan of treatment that we know of that has any effect upon it; we have a patient that has a fight before him and the best thing is to get him ready for it. In dealing with typhoid, antipyretics are depressing. All these remedies should be let alone. None can be used with advantage, and they give your patient a less chance when it comes to the crisis. The cold water, I think, is the best remedy that you can use, with one exception. Frequently you will have a patient that will not stand the cold water. With these patients you can use hot water, or some other method. These means of reducing the temperature are the best I know of. After the patient has passed well into the course of the fever and gets into the irritable condition, when they have a cold perspiration, my plan is to give atropine in full doses, and repeat in full doses three times a day. The skin does not perspire so freely. I never use medicine by the stomach, as far as I can possibly avoid it. I use strychnine three or four times a day in full dose, the same as in surgical operations, and be guided by the effect. The remedies should be used entirely for the effect. No case of typhoid fever is a rule for treatment. When you see them, not only see the fever, but recognize the temperament of the patient. I think sometimes that the thermometer is the worst thing we could have, as it often causes us to pay more attention to the temperature than to the treatment of the general condition of the patient."

DR. HURT: "I think that typhoid fever should be recognized as a product of germ or bacteria, and the sooner we recognize the case and make an attack on the condition of things, the better we are able to control the fever, and the better we are apt to hold up the strength of the patient, if the fever should be of long duration. It is not hard to arrive at a diagnosis in the regular forms, but in some cases they are so masked as to make it difficult. For instance, the patient has the low morning temperature and high evening temperature, without any intermission of the fever at all, and it will run on this way for eight days; you may give mercury and quinine, and on the eighth day find the patient with a normal temperature and seemingly all right and getting well, and then wait twelve hours and discover a rise of temperature, and have to wait from four to eight weeks to get rid of the fever. I believe that to make an attack on typhoid fever you must recognize the dry, brown tongue, with red edges, headache of constipation, to begin giving a good dose of mercury to arouse the secretions and to get the bowels empty. You have then placed the patient where you will take up the line of treatment which will meet the full condition. If in some cases after giving the mercury we fail to get any moisture on the tongue, I have found the patient in condition for the use of salol and phenacetin, but in the early stages of the fever these do not bring the fever down, unless in large doses, and I would say that my use of these is limited to small doses. I do not believe it is safe to give large doses in hardly any case. I believe that two grains will control better than five grains, as five grains will reduce to below normal, and then the nervous rigor comes on and the temperature rises again. I believe that salol has sufficient antipyretic effect in these instances if combined with phenacetin. When placed in the bowels two to four hours we are able to keep down fermentation and gaseous condition of the bowels. In this way we control the fever by keeping the causes inert. As the patient advances to the second, third, or fourth week, we have to be careful about the use of any antipyretic. I am inclined to believe that the use of aconite in small doses, combined with alcohol, is an excellent thing. I remember a case two years ago which ran twenty-four days. I kept up the continuous use of aconite during the entire course of the fever, and held the temperature to 101 and 102, but would leave off for a day or so and the temperature would go to 104, and then gave phenacetin but the patient would have rigor. As to those cases in which delirium occurs, I think it is highly important to pay special attention to the nervous system, and to do that we have the different nervous sedatives. I use the bromide of potash and extract of cannabis indica. While it tends to hallucination, yet also has a hypnotic tendency. As to hemorrhage, I have had the misfortune, in my experience to see ten or twelve cases of violent hemorrhages from the bowels. My experience has extended elsewhere, like Dr. Duncan's, and several have died. This need of restraining hemorrhage from ulcerated bowels demands all of a man's tact and energy, and with all that he is liable to make a sad failure. One case of a boy twenty-five years of age, bled profusely in the fifth week of the fever, and I used Monsel's solution in glass of water and forced up the rectum. It did good, as he had no further movement from his bowels, but on the fifth day after this injection he passed a coagulum of blood about six inches long. I thought the pressure of the drug arrested the hemorrhage and he got well. Another case had a discharge on the fifteenth day and died in a few hours. I saw a negro boy about eighteen years of age, and his first symptom which commanded attention was hemorrhage. He had been feeling bad for a day or two, and in one night had six or eight bloody actions. I used deodorized tincture of opium. and he got all right, but afterwards I gave him a dose of oil for his fever and he bled to death. I think stimulants in typhoid fever are often too early begun. As a rule, the majority of physicians begin the stimulants before it is necessary. As long as the patient can take nourishment and keep up, I do not think you should begin stimulating early but should hold this in reserve."

W. L. CHAMPION, M.D.,
Secretary.

---

### APPENDICITIS AND LIFE INSURANCE.

In the *Medical Examiner*, the editor, Dr. Wells, discusses Appendicitis in its relation to life insurance, with these conclusions:

The question of the acceptance of a case of an applicant who has ever had appendicitis is, therefore, a serious one. We are not favorably disposed to the acceptance of one who has had several attacks, it matters not how long the intervals between them, and who had never been operated for a radical cure. The cause of this condition may not be a mere inflammation, but cases have been known to occur in phthisical patients, that is, consumptive patients, and this may be the origin of the whole trouble. Of course in such a case we would not think of accepting or issuing any insurance. The fact that it may recur a great many times, even as you will see from one to two hundred times, will make the case absolutely uninsurable in any event. Some companies have a rule that if a party has had appendicitis once, presumably of the so-called catarrhal variety, he is insurable after five years, and if he has been radically cured by an operation, three years must elapse; but even this rule has its doubtful side, because, as we have seen, all diagnoses of appendicitis have heretofore been in many cases of very little value among physicians at large. It is only within a very few years that the diagnosis of appendicitis has been placed upon anything like a true basis, thanks to the American surgeon.

There is no general rule among companies, but the writer, from these studies, would most assuredly advise exceedingly great caution in the acceptanee of risks of this kind.

